# The Toll-like receptor 5 agonist entolimod suppresses hepatic metastases in a murine model of ocular melanoma via an NK cell-dependent mechanism

**DOI:** 10.18632/oncotarget.6500

**Published:** 2015-12-08

**Authors:** Hua Yang, Craig M. Brackett, Vanessa Marie Morales-Tirado, Zezhong Li, Qing Zhang, Matthew W. Wilson, Camille Benjamin, Wayne Harris, Edmund K. Waller, Andrei V. Gudkov, Lyudmila G. Burdelya, Hans E. Grossniklaus

**Affiliations:** ^1^ Department of Ophthalmology, Emory University, Atlanta, GA, USA; ^2^ Department of Cell Stress Biology, Roswell Park Cancer Institute, Buffalo, NY, USA; ^3^ Department of Ophthalmology, Hamilton Eye Institute, The University of Tennessee Health Science Center, Memphis, TN, USA; ^4^ Deparment of Hematology and Medical Oncology, Winship Cancer Institute, Emory University, Atlanta, GA, USA; ^5^ Cleveland BioLabs, Inc., Buffalo, NY, USA

**Keywords:** TLR5, entolimod, uveal melanoma, liver metastases

## Abstract

Uveal melanoma (UM) is the most common primary cancer of the eye in adults and progresses to metastatic disease predominantly of the liver in ∼50% of patients. In these cases, life expectancy averages just 9 months due to the lack of effective treatment options. The Toll-like receptor 5 (TLR5) agonist entolimod (former name CBLB502) rapidly activates TLR5-NF-κB signaling in hepatocytes and suppresses growth of both TLR5-expressing and non-expressing tumors in the liver through mobilization and activation of innate and adaptive immune mechanisms. The goal of this study was to explore the potential of entolimod as an immunotherapeutic agent against hepatic metastasis of UM using the TLR5-positive B16LS9 mouse model of ocular melanoma. Mice were given seven subcutaneous injections of vehicle or entolimod given 72 h apart started one day before, on the same day or three days after intraocular injection of B16LS9 cells. All tested regimens of entolimod treatment resulted in significantly reduced B16LS9 metastasis to the liver. Entolimod induced mobilization of natural killer (NK) cells to the liver and stimulated their maturation, differentiation and activation. Antibody-mediated depletion of NK cells from mice abrogated entolimod's antimetastatic activity in the liver and eliminated the entolimod-elicited *in vitro* cytotoxic activity of hepatic lymphocytes against B16LS9 cells. These results provide pre-clinical evidence of entolimod's efficacy against hepatometastasis of UM and support its further development as an anticancer immunotherapeutic drug.

## INTRODUCTION

Uveal melanoma (UM) is the most common primary cancer of the eye in adults, with 2,000–2,500 new diagnoses per year in the United States. Primary UM tumors are usually treated with surgery (plaque brachytherapy, enucleation of the tumor-bearing eye), radiation therapy and/or thermotherapy [1–3]. Despite these treatments, about 50% of all UM cases progress to metastatic disease within 15 years of diagnosis. Most metastasis of UM (80-90%) occurs in the liver [4, 5]. UM patients with a “class 2” gene expression signature have an extremely poor survival prognosis due to development of detectable metastases in the liver within 3 years of primary tumor diagnosis [6, 7]. There are currently no effective treatments for metastatic UM and there is an average life expectancy of 9 months from the time of detection of metastases.

Anticancer immunotherapy is emerging as a powerful cancer treatment strategy, especially for patients with recurrent and/or metastatic disease [8, 9]. One approach to enhancing anti-tumor immune responses is pharmacological activation of different Toll-like receptors (TLRs), which play a key role in activation of the innate immune system through their recognition of specific pathogen-associated molecular patterns (PAMPs) [10, 11]. Upon interaction with their ligand (the natural PAMP or an engineered agonistic agent), TLRs engage a set of adaptor proteins through homophilic interaction of their Toll/IL-1 receptor (TIR) domains. This triggers downstream signaling cascades leading to activation of the transcription factor NF-κB. Activated NF-κB stimulates expression of a number of target genes, including several encoding pro-inflammatory cytokines and chemokines that promote mobilization and activation of neutrophils, Natural Killer (NK) cells and dendritic cells. These cells play essential roles in innate immune responses and are also involved in development of adaptive immune responses through stimulation of T-cell activation. Thus, recognition of PAMPs by TLRs is a key element for induction of inflammatory responses, as well as for instruction of immune responses against pathogens and tumor cells.

The powerful immunoregulatory activity of TLRs has led to consideration of agonists of several TLRs (e.g., TLR7, TLR9) as anti-cancer immunotherapeutics [12–14] and development of novel TLR agonist-based therapies aimed at stimulating host anti-tumor immune responses [15, 16]. Our work has focused on the immunotherapeutic potential of TLR5 stimulation using the novel recombinant protein drug entolimod, which is a pharmacologically optimized derivative of the natural TLR5 ligand, bacterial flagellin. Details of the high affinity interaction between flagellin/entolimod and TLR5 were revealed by solving the crystal structure of the complex [17], and the downstream signaling cascade resulting from this interaction is well-established [10, 11]. Compared to other TLR family members, TLR5 has significant advantages in terms of safety for potential clinical targeting due to the specific pattern of TLR5 expression by mammalian tissues and the nature of cytokines induced following TLR5 stimulation. Unlike some other TLRs (e.g., TLR4), TLR5 signaling does not result in a highly inflammatory, potentially dangerous “cytokine storm.” Entolimod was originally described as a radiation countermeasure based on its ability to reduce radiation damage to normal tissues (but not tumors) and improve their regeneration in mice and non-human primates through TLR5-dependent activation of NF-κB [18, 19]. The same TLR5-dependent cascade of molecular events promoting cytokine expression and immune cell stimulation provided the foundation for exploration of TLR5 agonists as anticancer immunotherapeutics [20].

Potent antitumor efficacy of flagellin and its derivatives such as entolimod was demonstrated in syngeneic and xenogeneic mouse models of TLR5-expressing tumors of various origins and was shown to involve stimulation of immune responses [18, 20–24]. In addition, entolimod was found to suppress growth of both TLR5-positive and TLR5-negative tumors residing in the liver through activation of TLR5 on hepatocytes. Syngeneic liver metastatic models of colorectal CT26 and mammary 4T1 cancer were used to show that the metastasis suppressive effects of entolimod involved mobilization to and activation of neutrophils and NK cells in the liver [20]. These data led us to hypothesize that entolimod might effectively suppress hepatic metastasis associated with UM. We tested this hypothesis in a mouse model of UM in which orthotopic intraocular B16LS9 UM tumors spontaneously metastasize to the liver. Our results indicate that systemic entolimod treatment led to a significant reduction in the number of metastatic nodules in the livers of mice in this model. This entolimod mediated antimetastatic effect was associated with increased blood-borne homing, maturation and activation of NK cells in the liver, and was abrogated when mice were depleted of NK cells before entolimod treatment. Therefore, this study provides confirmation of entolimod's efficacy in suppressing hepatic metastasis through immune stimulatory mechanisms involving NK cells and suggests that entolimod could be used as a new therapy to treat metastatic UM.

## RESULTS

### Entolimod treatment suppresses hepatic metastasis of ocular melanoma

**Figure 1:**
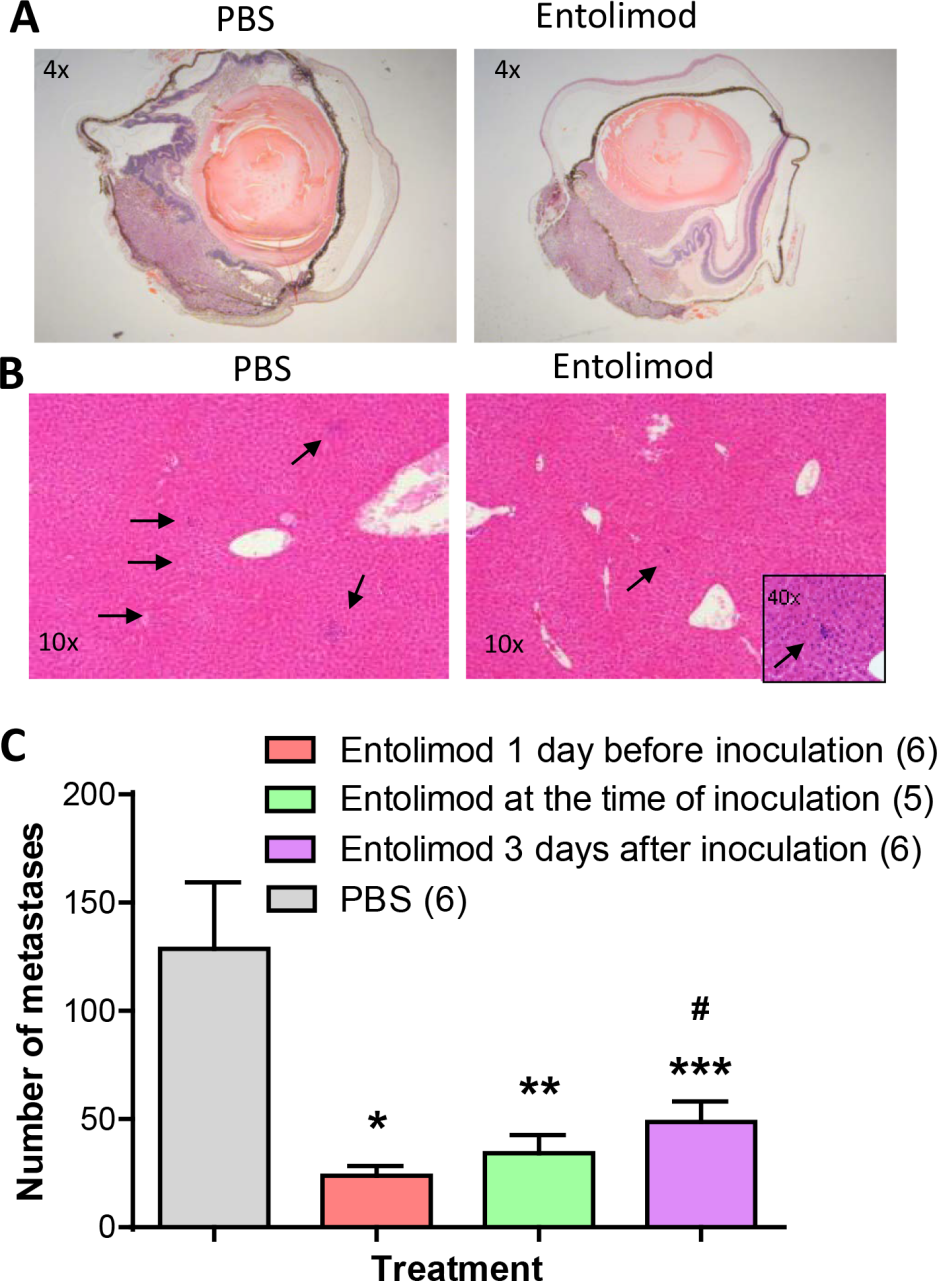
Effect of entolimod treatment on liver metastasis of B16LS9 UM tumors following enucleation of the tumor-bearing eye (**A**) Representative pictures (4 × magnification) of H&E-stained sections of eyes with intraocular tumors from mice injected with vehicle (PBS-T) or entolimod. Mice received two injections of entolimod (1 μg/mouse, s.c., on the day of tumor cell inoculation and 72 h later) or vehicle control (PBS). Eyes were enucleated from mice seven days after B16LS9 cell inoculation. (**B**) Representative photographs of H&E-stained sections of livers collected from mice 21 days after B16LS9 cell inoculation in the eye. Seven injections of entolimod (1 μg/mouse, s.c., with 72 h intervals starting on the day of tumor cell inoculation) or vehicle control (PBS) were performed. Arrows point to metastases. (**C**) Quantitative analysis of hepatometastases was performed by counting metastatic nodules in three H&E-stained sections of each animal's liver. The per-group mean number of metastases per liver ± SEM is shown with the number of analyzed mice per group indicated in parentheses. Statistical significance was determined by Student's *t*-test: *p = 0.0069; **p = 0.0233; and ***p = 0.0316 compared to vehicle group; ^#^*p* = 0.0395 compared to entolimod treatment starting one day before inoculation.

In this study, efficacy of entolimod in suppressing liver metastasis was tested in the well-established and previously described B16LS9 syngeneic UM mouse model [25]. C57BL/6 mice were inoculated with B16LS9 tumor cells in the choroid of the right eye. Groups of mice (*n* = 10 mice/group) were treated with seven s.c. injections of vehicle (phosphate-buffered saline/0.1% Tween 80; PBS-T) or entolimod (1 μg/mouse) given 72 h apart. The vehicle treated group was treated beginning one day before tumor cell inoculation. The three entolimod-treated groups were treated beginning (i) one day before, (ii) on the same day as, or (iii) three days after tumor cell inoculation. Seven days after tumor cell inoculation, the tumor-bearing eye was removed and intraocular tumor growth was histologically confirmed in all mice (Figure 1A). Although B16LS9 cells express functional TLR5 and respond to entolimod treatment with NF-κB activation (indicated by p65 translocation to the nucleus 30 min after *in vitro* treatment with entolimod (Supplementary Figure S1)), there was no significant difference in the size of primary melanomas in the eyes of entolimod-treated (all three treatment schedules) versus vehicle-treated mice as measured on Day 7 after tumor cell administration (Figure 1A, Supplementary Figure S2A). During following 3 weeks of observation, about 3–5 mice in each group developed lung metastases and died on days 15–20 after tumor cell inoculation independently on entolimod treatment (data not shown). On Day 21 after tumor cell inoculation, the surviving mice (*n* = 5–7 per group) were sacrificed to evaluate the effect of entolimod treatment on livers and lung metastasis of B16LS9 tumors in this model. The number of lung metastases was determined in one section from each lung after hematoxylin and eosin staining. There was not a significant difference in the number of lung metastases in entolimod treated (all three treatment schedules) versus vehicle-treated mice (*P* > 0.05, Supplementary Figure S2B). In contrast, the number of metastases per liver was significantly lower in all entolimod treated groups compared to the vehicle treated control group (Figure 1B, 1C). The lowest number of hepatic metastasis was observed in the group of mice given entolimod beginning one day before tumor cell inoculation (23.83 ± 11.37), slightly more metastases were observed in the group given entolimod beginning on the day of inoculation (34.2 ± 18.95), and the highest number of hepatometastases was found in the group that started entolimod treatment three days post-inoculation (48.83 ± 23.24). The only statistically significant difference between entolimod-treated groups was between the lowest (treatment initiation one day before) and the highest (treatment initiation three days after) numbers of metastases (*p* < 0.05). There was no general toxicity observed in mice due to entolimod treatment (no weight loss, mortality). Apart from the presence of B16LS9 metastases, the hepatic tissue from all mice exhibited normal morphology without any signs of toxicity (no blood vessel damage, necrosis or vacuolar changes in hepatocytes) at this time-point (data not shown). These results demonstrate specific antitumor activity of systemically administered entolimod against UM tumor growth in the liver.

### Entolimod treatment stimulates blood-borne homing of NK cells to the liver

**Figure 2:**
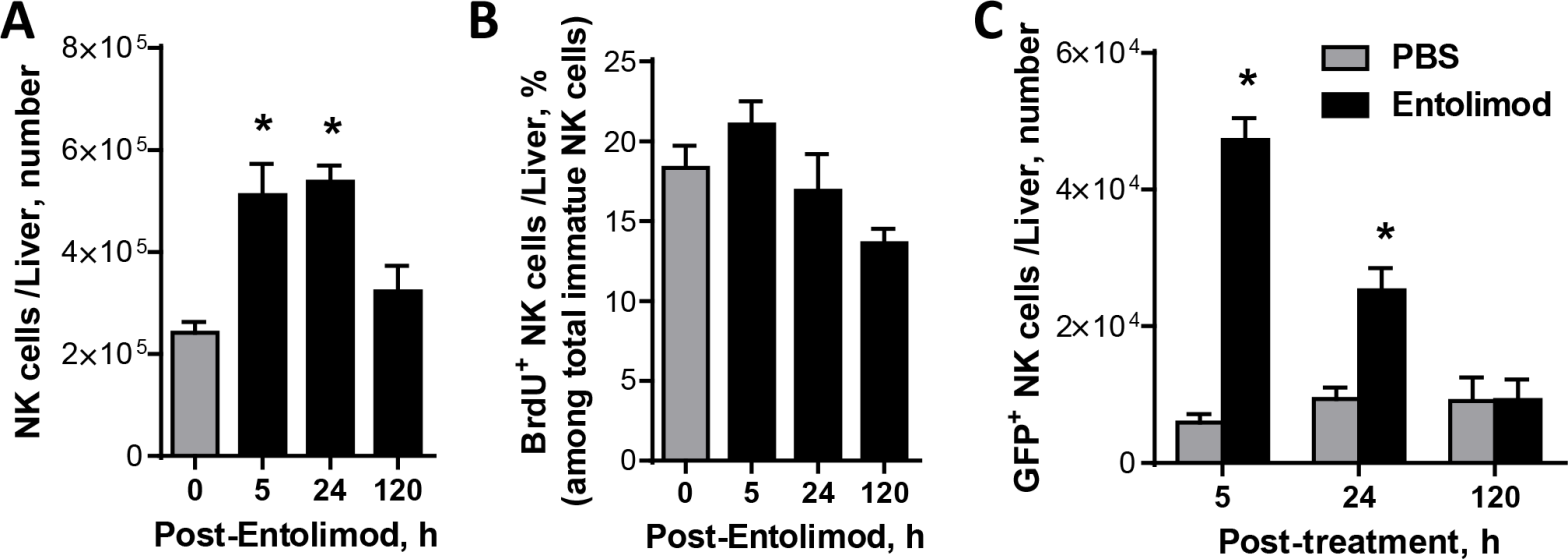
Entolimod-elicited NK cell response in the livers of naïve non-tumor-bearing mice (**A**) Absolute numbers of NK cells in livers of intact (0 h time point) and entolimod-treated C57BL/6 mice. Liver cells were harvested from mice 5, 24 or 120 h after receiving a single s.c. injection of 1 μg entolimod by *in vivo* perfusion and *in vitro* digestion. The total cell population was stained with a cocktail of antibodies against CD45, CD3ε, and NK1.1 in order to quantify NK cells (CD45^+^ CD3ε^−^NK1.1^−^) by FACS. (**B**) Percentage of BrdU positive NK cells among total NK cells in the liver. Livers were collected as in (A) except that mice were injected with 1 mg BrdU i.p. 2 hours before liver cells were harvested. Staining was performed using the antibody cocktail described in (A) plus anti-BrdU antibody. (**C**) Absolute numbers of GFP^+^ NK cells in livers of mice given GFP-expressing splenocytes by i.v. adoptive transfer immediately before treatment with vehicle (0 h) or entolimod as described in (A). Livers were collected at the indicated time points as in (A) and total liver cells were analyzed by FACS. Transferred NK cells were defined as CD45^+^ GFP ^+^ CD3ε^−^ NK1.1 +. For A–C, mean ± SEM values are shown for groups of 5 mice. (*) Student *t*-test shows significant difference from untreated groups (0 h time point), *P* < 0.05.

The previous studies in syngeneic liver metastatic models of colorectal CT26 and mammary 4T1 cancer demonstrated anti-metastasis activity of entolimod that involved mobilization and activation of NK cells to the liver [20]. Therefore, we investigated the role of NK cells in the activity of entolimod in the UM model. In mice given a single s.c. injection of entolimod, the number of total NK cells in the liver was significantly increased at 5 h post-treatment and remained elevated for at least 24 h before returning to normal levels by 120 h (Figure 2A). Since the liver contains both resident and infiltrating lymphocytes including NK cells in both populations with potential antitumor function [26–28], we next sought to determine whether the increased number of NK cells in the liver was due to proliferation of resident NK cells or blood-borne homing of additional NK cells to the liver. To assess NK cell proliferation, naïve mice were injected s.c. with vehicle or entolimod (1 μg/mouse) followed by BrdU as described in Materials and Methods. FACS analysis performed at 5, 24, and 120 h post-treatment showed a similar level of NK cell proliferation in the livers of vehicle and entolimod-treated mice (∼20% of total NK cells in the liver incorporated BrdU) (Figure 2B). This led us to hypothesize that entolimod stimulates blood-borne homing of NK cells to the liver. To test this hypothesis, we performed a homing assay in which GFP-expressing splenocytes from naïve C57BL/6 mice were transfused via an intravenous injection into naïve C57BL/6 mice immediately prior to entolimod or vehicle treatment (single s.c. injection). Livers were collected and processed for FACS analysis (including staining with antibodies against NK cell-specific markers) at different times post-treatment. This revealed a significant increase in adoptively transferred (GFP^+^) NK cells in the liver at 5 h after entolimod treatment compared to vehicle treatment (Figure 2C). Levels of these cells remained elevated for at least 24 h before returning to control levels by 120 h post-treatment. These results indicate that entolimod treatment of naïve mice stimulates homing of NK cells to the liver via a blood-borne mechanism.

### Entolimod stimulates hepatic NK cell maturation, differentiation, and activation

**Figure 3:**
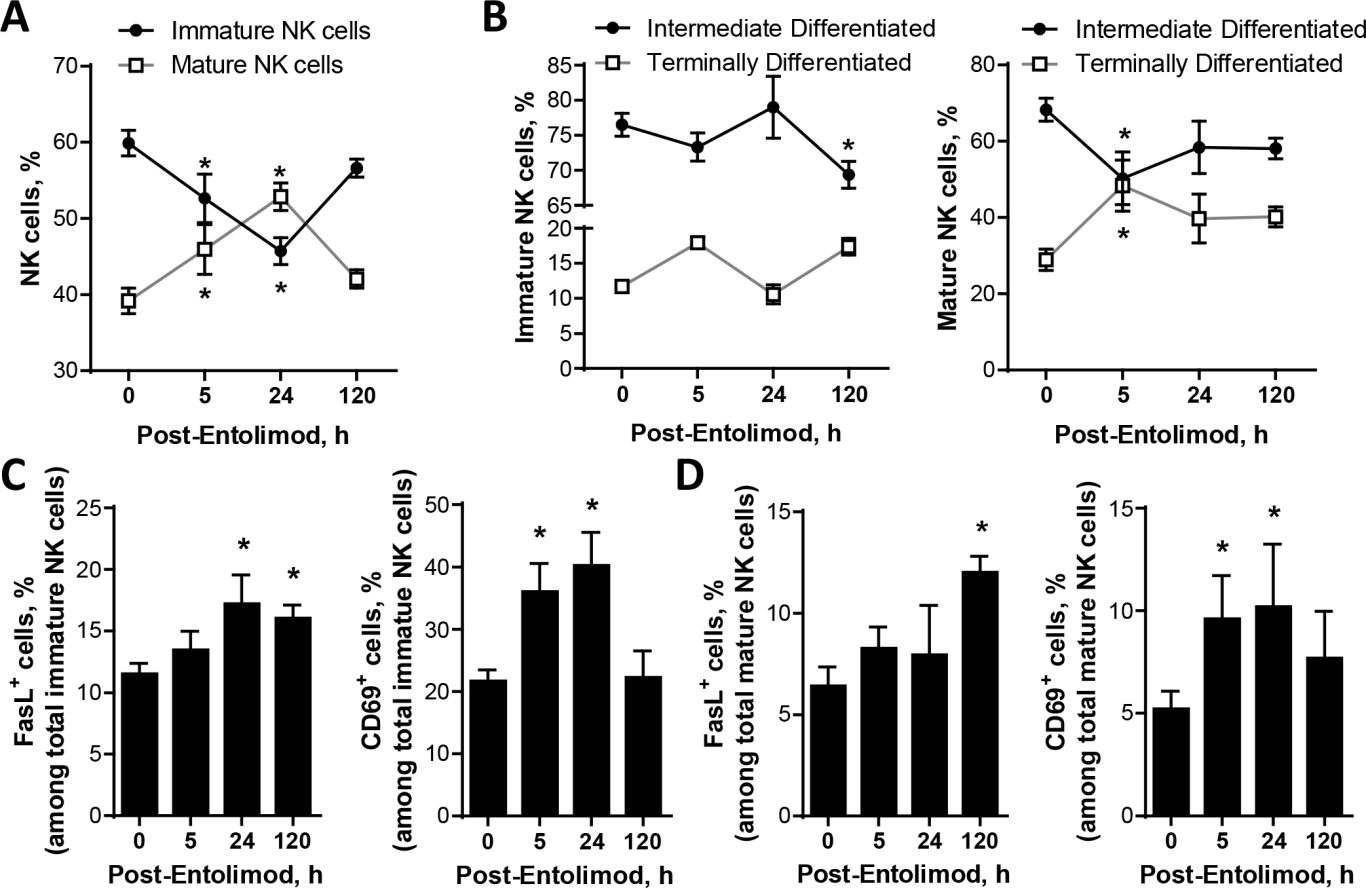
Effect of entolimod treatment on the maturation and activation status of NK cells in the livers of non-tumor-bearing mice Liver cells were collected at the indicated time points (*n* = 6–30 mice/time point) after entolimod treatment (single s.c. injection of 1 μg/mouse) by *in vivo* perfusion and *in vitro* digestion to assess the frequency of mature, differentiated, and activated NK cells (CD45^+^ CD3ε^−^NK1.1^+^) by FACS (mean ± SEM). Data from a vehicle-treated group is shown as the “0” h time point. (**A**) Mature NK cells were defined by acquisition of CD49b expression. (**B**) Intermediate differentiation status was defined as CD11b^+^ CD43^−^ and terminal differentiation status by CD11b^+^ CD43^+^. (**C–D**) Activated NK cells were defined by expression of FasL or CD69. (*) Student *t*-test shows significant difference from untreated groups (0 h time point), *P* < 0.05.

Immature NK cells within the liver undergo maturation and differentiation following viral infections [29, 30]. Using naïve non-tumor-bearing mice, we sought to determine whether entolimod treatment stimulates a similar pattern of hepatic NK cell maturation and differentiation by following the acquisition of specific markers of NK cell maturation [CD49b (DX5)] and differentiation (CD11b and CD43) by cells in the hepatic NK lineage (NK1.1^+^CD3ε^−^) using FACS as described previously [29–31]. Within five hours after s.c injection of entolimod (1 μg/mouse), the proportion of NK cells in the liver with an immature phenotype decreased and a corresponding increase in NK cells with a mature phenotype was observed (Figure 3A). This shift continued up to 24 h post-treatment, but then normalized by 120 h post-treatment. Mature, but not immature, NK cells acquired a terminally differentiated phenotype within 5 h of entolimod treatment, which was observed for at least 5 days after entolimod treatment (Figure 3B).

Having shown that entolimod promotes maturation of NK cells in the liver, we next evaluated the effect of entolimod on the activation status of both immature and mature NK cells in the liver as indicated by Fas ligand (FasL) and CD69 expression. Within the immature NK cell population, the proportion of FasL-expressing cells increased by 24 h after entolimod treatment and remained elevated for at least 120 h (Figure 3C). Among mature NK cells, the proportion of FasL-expressing cells was not elevated until 120 h post-entolimod (Figure 3D). The proportion of CD69^+^-expressing cells within both mature and immature NK cell populations in the liver was elevated at 5 h and 24 h after entolimod treatment (Figure 3C, 3D). Taken together, these findings demonstrate that entolimod treatment stimulates maturation, differentiation and activation of NK cells within the liver.

### Entolimod stimulates NK cell development and maturation in the livers of UM tumor-bearing mice

**Figure 4:**
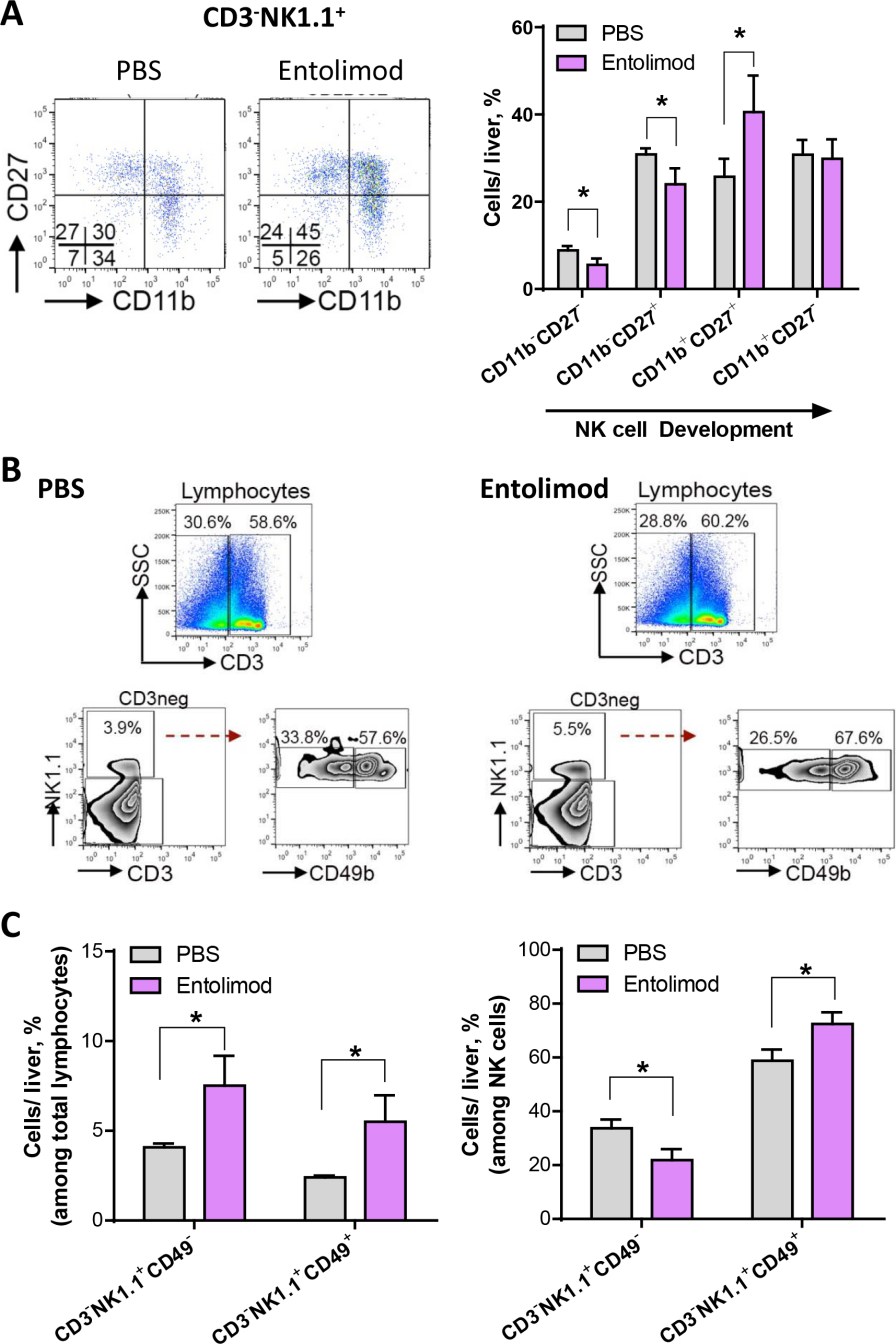
Entolimod induces development and maturation of NK cells in the liver of UM tumor-bearing mice (**A**) Hepatic lymphocytes were isolated from PBS-T (vehicle) and entolimod-treated mice 21 days after B16LS9 tumor cell inoculation using Lymphocyte M separation media. Entolimod (1 μg/mouse) or PBS-T vehicle was administered by s.c. injection every 3 days starting on the day of tumor cell inoculation (7 injections total). Representative images of FACS analysis of CD3^−^NK1.1^+^ cells divided based on their expression of CD27 and CD11b. Four distinct populations, CD27^+^ CD11b^−^, CD27^+^ CD11b^+^ (double positive), CD27^−^CD11b^+^ and CD27^−^CD11b^−^ (double negative) were examined. The bar graph on the right shows the cumulative results in each treatment group, mean ± SEM, *n* = 3/group. (*) Student's *t*-test shows significant difference between PBS-T- and entolimod-treated groups, *P* < 0.05. (**B**) Representative images of FACS analysis and percentage of NK cells gated as CD3^−^NK1.1^+^ and then sub-gated based on CD49 expression. Red dashed arrow indicates the separation into CD49b^−^ and CD49b^+^ populations. SSC, side scatter. (**C**) The average percentage (mean ± SEM) of CD49b^−^ and CD49b^+^ cells among total hepatic lymphocytes (CD3^−^) (left) and among CD3^−^NK1.1^+^ NK cells (right) per liver was calculated for groups of mice treated as in (A). *n* = 3 mice/group. (*) Student *t*-test shows significant difference between PBS-T- and entolimod-treated groups, *P* < 0.05.

Our finding that entolimod treatment results in increased percentage of mature, differentiated and activated NK cells in the livers of naïve C57BL/6 mice suggests a role for NK cells in the antimetastatic effects of entolimod observed in the B16LS9 UM model. First we investigated the effect of entolimod on NK cell development in the liver of mice with metastatic B16LS9 tumors. Four distinct stages of NK cell development were defined based upon surface expression of CD27 and CD11b that starts with low expression of both CD27 and CD11b and leads through the following stages: high CD27 and low CD11b expression, then high CD27 and high CD11b and then low CD27 and high CD11b, as previously reported^23^. This development program is associated with progressive acquisition of effector function. Figure 4A shows representative FACS plots for expression of CD27 and CD11b within the hepatic NK cell lineage isolated from one mouse from each group treated with vehicle or entolimod every 3 days starting on the day of B16LS9 tumor cell inoculation (7 injections total). Entolimod treatment significantly reduced the less developed NK cell populations (CD27^−^CD11b^−^ and CD27^+^CD11b^−^). In addition, entolimod treatment significantly increased the more developed double positive CD11b^+^CD27^+^ population (*p* < 0.05), which is associated with a cytotoxic phenotype. Entolimod had no effect on the most highly developed CD27^−^CD11b^+^ NK cell population, which is thought to lack effector function. Collectively, these results demonstrate that the entolimod-induced NK cell response in the liver is associated with an increase in the development of NK cells into a more mature phenotype equipped with effector function.

Next we assessed the effect of entolimod treatment on the maturation status of NK cells in the livers of mice bearing UM liver metastases 21 days after B16LS9 tumor cell inoculation. Figure 4B shows representative FACS plots (from one vehicle-treated mouse and one entolimod-treated mouse) for the hepatic NK lineage (NK1.1^+^CD3ε^−^) with analysis of maturation marker CD49b, as described above for non-tumor-bearing mice with percentage indicating the proportion of mature and immature NK cells among total liver lymphocytes and within NK cell population. Figure 4C provides the quantitative results of the analysis for groups of 3 mice treated with either vehicle or entolimod (mean ± SEM). The average percentage of NK cells among total liver lymphocytes was significantly increased from 4.1% in the vehicle-treated group to 7.5% in the entolimod-treated group (Figure 4C, left). Within the total NK cell population in the liver, entolimod treatment led to a reduced proportion of immature NK cells (from 33.7% to 21.8%) and a concomitant increase in the proportion of mature NK cells (from 58.8% to 72.4%) (Figure 4C, right).

**Figure 5:**
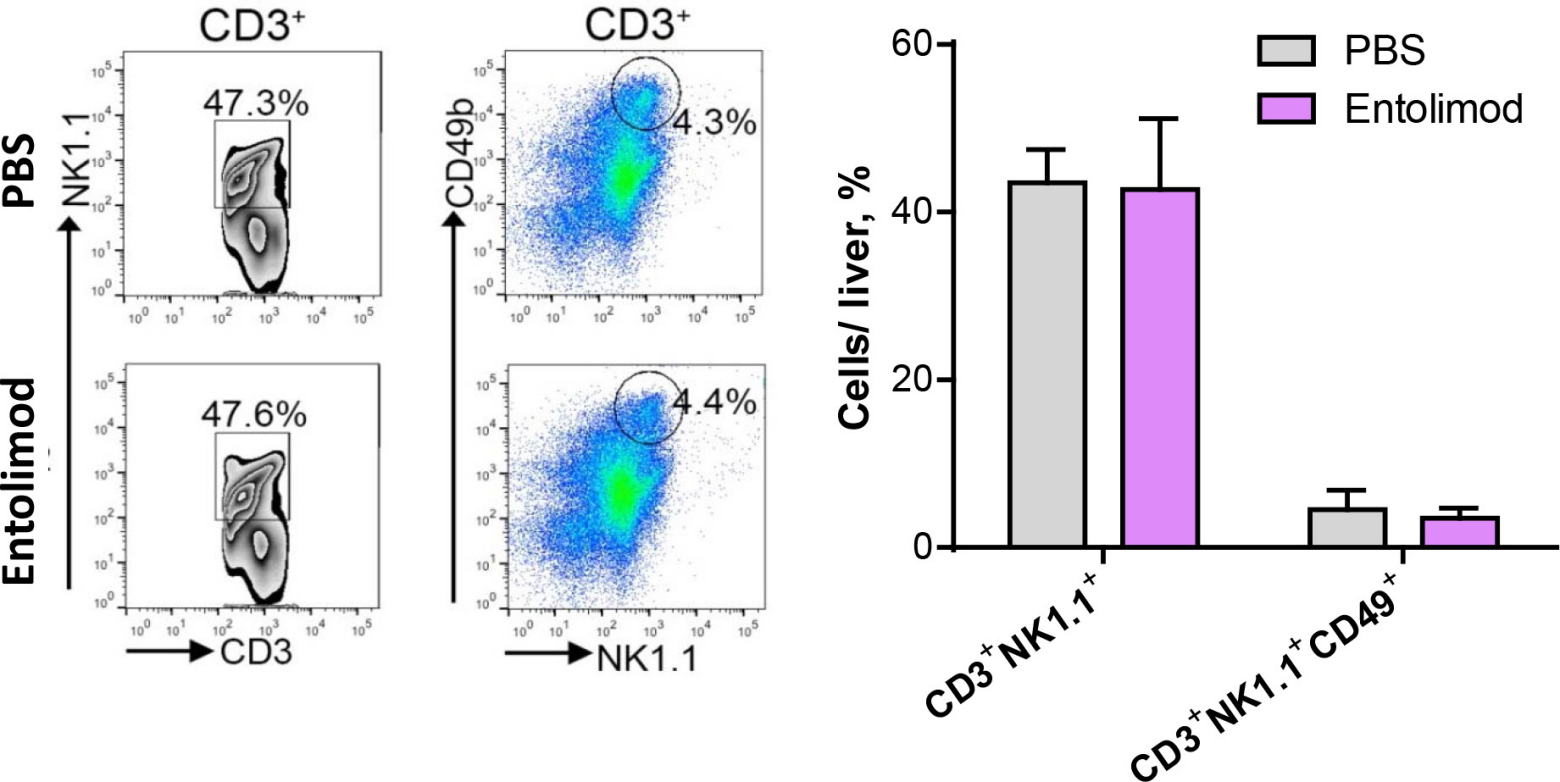
Entolimod has no effect on maturation of NKT cells in the liver (**A**) Representative images of FACS analysis of CD3^+^ lymphocytes analyzed for NK1.1 and CD49b expression. *Left column*, CD3^+^ cells were plotted against NK1.1. The double positive population was gated. *Right column*, CD3^+^ cells were examined for NK1.1 and CD49b positivity, represented by the circle. The bar graph on the right shows the cumulative results for each group, mean ± SEM, *n* = 3/group. Student's *t*-test showed no significant difference between PBS-T- and entolimod-treated groups.

In addition to NK cells, NKT cells also reside in the liver and can have anti-tumor functions [26, 32]. Since entolimod treatment was previously shown to increase the absolute number of NKT cells in the livers of naïve mice [20], we sought to investigate whether entolimod affects NKT cells in UM tumor-bearing livers. NKT cells were defined as CD3^+^NK1.1^+^ cells and further subdivided into CD49b^+^ mature and CD49b^−^ immature populations. FACS analysis with antibodies against the appropriate markers was performed on total liver cells isolated from UM bearing mice 21 days after B16LS9 tumor cell ocular inoculation and treated with entolimod/PBS every 3 days starting on the day of tumor cell inoculation (Figure 5). In contrast to what was shown in the livers of naïve mice, entolimod treatment had no effect on the percentage of NKT cells or their maturation status in the livers of UM tumor-bearing mice. Therefore, in mice bearing UM tumors, entolimod specifically induces mobilization to the liver and maturation of NK cells without having any significant effect on NKT cells in the liver.

### NK cells mediate the anti-tumor activity of entolimod against UM liver metastases

**Figure 6:**
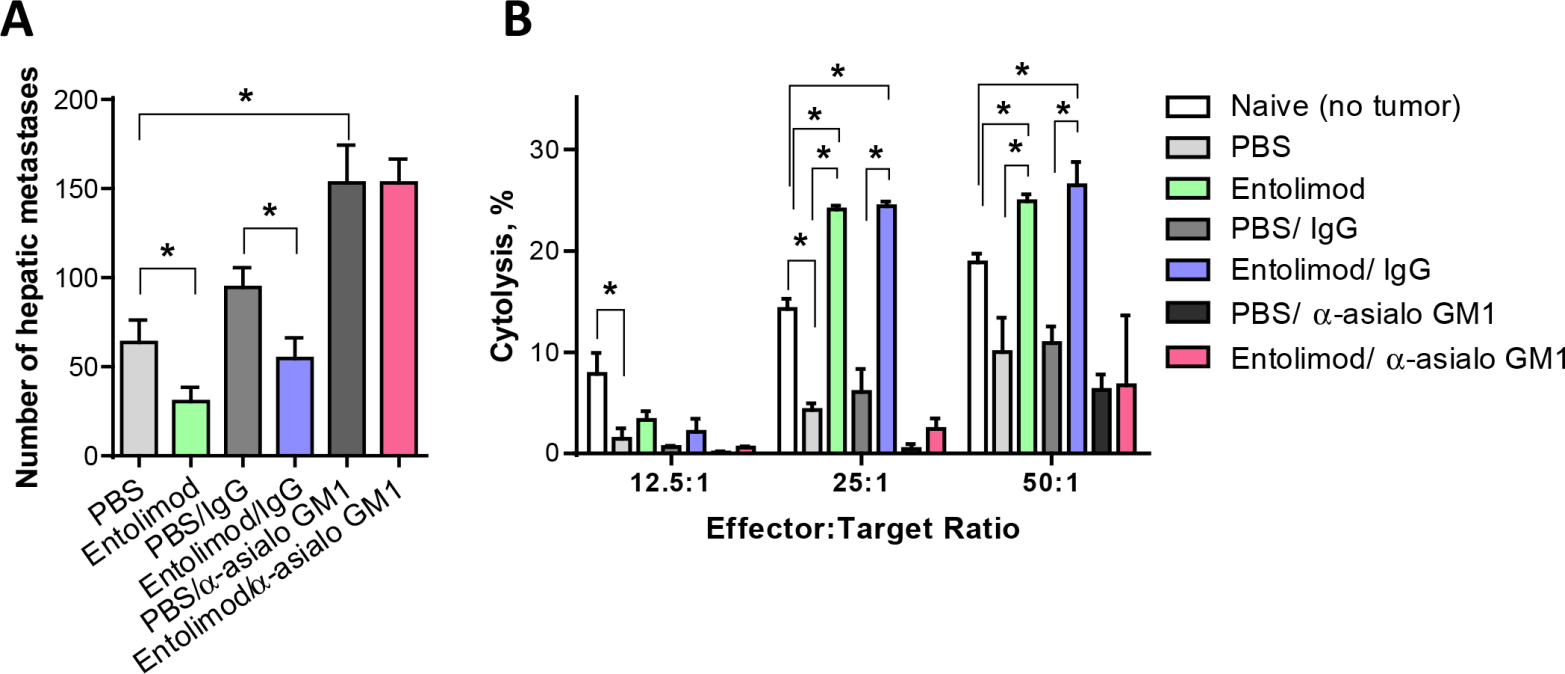
Entolimod antitumor activity of against UM metastases is NK cell-dependent (**A**) The number of hepatic metastases per liver (mean ± SEM, *n* = 6 mice/group) on Day 21 after B16LS9 tumor cell inoculation is shown for groups treated with vehicle (PBS-T), entolimod, PBS-T with control IgG antibody, entolimod with control IgG antibody, PBS-T with anti-asialo GM1 antibody or entolimod with anti-asialo GM1 antibody (see text for details on treatments). Asterisks indicate statistically significant differences (*P* ≤ 0.05) between groups determined by Student's *t*-test. The difference between anti-asialo GM1 antibody treated groups and any other group was statistically significant (*p* < 0.001). (**B**) *In vitro* cytotoxicity (CT) mediated by hepatic lymphocytes isolated from mouse livers on Day 21 after B16LS9 tumor cell ocular inoculation (mean ± SD, 6 mice/group). Mice were treated as described in (A). Hepatic lymphocytes were incubated with B16LS9 target cells at effector-to-target cell ratios of 12.5:1, 25:1 and 50:1. (*) Student *t*-test shows significant difference between the indicated groups, *P* < 0.05.

To determine the relevance of the observed NK cell response in the livers of entolimod-treated mice for the drug's antitumor activity, systemic administration of anti-asialo GM1 antibody was used to deplete NK cells in the context of the UM model. Mice were injected (i.p.) with anti-asialo GM1 antibody (or isotype matched control antibody) one day before intraocular B16LS9 tumor cell inoculation, on the day of inoculation, and then once every 4 days for a total of 7 injections. Entolimod or PBS-T was applied every 3 days starting on the day of tumor cell inoculation (7 injections total). FACS analysis of liver cell populations 21 day after B16LS9 tumor cell inoculation confirmed reduction of NK cell levels in the liver by > 80% following anti-asialo GM1 treatment (data not shown). Seven days after tumor cell inoculation, the tumor-bearing eye was enucleated. Histological examination of the section of the tumor bearing eye with the largest tumor area showed significantly increased intraocular tumor burden in mice depleted of NK cells by anti-asialo GM1 antibody in comparison to those given isotype control antibodies, regardless of whether the animals received entolimod treatment or not (Supplementary Figure S2A). This suggests the presence of a basal (entolimod-independent) level of innate NK cell-mediated antitumor activity against UM. Pathological examination of lungs collected from these mice 14 days after removal of the tumor-bearing eye (i.e., on Day 21 post-inoculation) showed that, with or without entolimod treatment, the mean number of metastases in the lungs was significantly higher in anti-asialo GM1 antibody-treated groups compared to control IgG-treated groups. Thus, growth of metastases in the lungs mirrored what was seen for primary tumors in the eye, implying the presence of systemic entolimod-independent innate NK cell activity against UM tumors (Supplementary Figure S2B). This was different in the liver, however, with entolimod treatment leading to a significant reduction in the number of metastatic nodules in mouse livers when comparing entolimod-treated and vehicle-treated groups given no antibody or control IgG antibodies (Figure 6A). The mean number of hepatic metastases was reduced from 63.63 ± 12.55 to 30.38 ± 8.05 by entolimod treatment in the absence of antibody (*P* = 0.04) and from 94.63 ± 10.94 to 54.63 ± 11.61 with IgG antibody treatment (*P* = 0.03). In contrast, there was no significant difference in liver metastasis between entolimod-treated and vehicle-treated groups when the mice were pretreated with anti-asialo GM1 antibody to deplete NK cells (Figure 6A). This indicates that the effect of entolimod on growth of B16LS9 metastases in the liver requires NK cells. Consistent with the indication of a basal level of entolimod-independent NK cell-mediated antitumor activity in all organs (see above), the number of liver metastases in NK cell-depleted mice (with or without entolimod treatment) was significantly higher than that seen in NK cell-sufficient mice (with or with entolimod treatment). Taken together, these findings demonstrate that the antimetastatic activity of entolimod in the liver is mediated by an NK cell-dependent mechanism.

As a direct assay of the antitumor activity stimulated by entolimod *in vivo*, we isolated the total lymphocyte population from livers of naïve mice or mice inoculated with B16LS9 tumor cells and treated with anti-asialo GM1 antibodies (or control IgG) and entolimod (or vehicle), as described above, and tested their cytotoxicity towards B16LS9 target cells *in vitro*. *In vitro* cytotoxicity (CT) assays were performed using different ratios of effector cells (isolated liver lymphocytes) to target cells (B16LS9 tumor cells). These assays revealed reduced CT activity of the total hepatic lymphocyte population in UM tumor-bearing mice compared with naive (non-tumor-bearing) mice which could be explained by immunosuppression developed in tumor-bearing animals (Figure 6B). With entolimod treatment, however, CT activity of liver lymphocytes against B16LS9 cells was not only restored, but significantly increased. This effect was not observed with hepatic lymphocytes isolated from anti-asialo GM1 antibody-treated mice (Figure 6B), demonstrating a requirement of *in vivo* entolimod-stimulated NK cells for B16LS9 cell elimination. As seen with hepatic lymphocytes, lymphocytes isolated from the spleens of naïve mice had a higher level of CT towards B16LS9 cells than those isolated from B16LS9 UM tumor-bearing mice (Supplementary Figure S3). In addition, as for liver lymphocytes, the CT of splenic lymphocytes was NK cell-dependent as indicated by the effect of anti-asialo GM1 antibody treatment. However, entolimod did not stimulate CT activity of splenic lymphocytes. This provides additional evidence of the tissue (liver) specificity of entolimod's activity leading to NK cell-mediated antitumor effects.

## DISCUSSION

The goal of this study was to assess the antitumor activity of the flagellin-based TLR5 agonist entolimod against hepatic micrometastases that spontaneously develop in mice with orthotopic uveal melanoma tumors in the eye. We used a well-established mouse model of ocular melanoma, B16LS9, which mimics the high frequency of liver metastasis observed in patients diagnosed with uveal melanoma [33, 34]. We previously reported that B16LS9 cells express low levels of MHC-I, but are highly sensitive to interferon α2b via activation of intrinsic hepatic NK cells [34]. Given our recent findings that entolimod activates NK cells within the liver leading to the development of powerful antitumor immune response and suppression of colorectal and breast liver metastases, we explored the ability of entolimod to suppress UM liver metastases in B16LS9 model through NK cell-dependent mechanism.

Our finding that B16LS9 cells respond directly to entolimod treatment by NF-κB activation (Supplementary Figure S1) indicates that entolimod might be expected to have some antitumor effect through TLR5 stimulation on B16LS9 tumor cells independently on TLR5 expression in tumor microenvironment, as was previously demonstrated in several other TLR5-positive tumor models [18, 21, 22]. However, entolimod did not show any antitumor activity against intraocular B16LS9 tumor growth in our experiments. This observation may be explained by the eye being an immune privileged site [35] or by the tested regimens of entolimod administration not being optimal for producing an antitumor effect in this particular tumor model. Similarly, B16LS9 lung metastases rapidly developed without any signs of tumor growth inhibition by entolimod treatment. In contrast to what was seen for B16LS9 tumor growth in the eye and metastasis to the lung, entolimod treatment did have a significant suppressive effect on metastasis to the liver. A course of seven subcutaneous entolimod injections given three days apart led to a significantly reduced number of hepatic metastasis developing from intraocular B16LS9 tumors regardless of when treatment was initiated (starting one day before, on the same day, or three days after tumor cell inoculation (Figure 1). Of the three tested regimens of entolimod treatment, initiation of treatment before tumor cell inoculation was most effective, followed by initiation of treatment on the day of inoculation, and then by initiation of treatment four days post-inoculation. These differences suggest development of an immunosuppressive mechanism during the initial stages of B16LS9 tumor establishment, growth and metastasizing. Overall, these data support our hypothesis that entolimod has a specific effect on the liver microenvironment due to stimulation of TLR5 on hepatocytes and that the modified microenvironment is not conducive for B16LS9 UM metastatic growth, presumably due to enhanced presence of immune cells with antitumor activity.

Given that NK cells play an important role in host immune responses against cancer [11] and in therapy-induced antitumor responses (as previously described in treatments with interferon α2b [34] and entolimod [36]), we sought to determine whether efficacy of entolimod against B16LS9 liver metastasis involves NK cells. The first step in this was to perform FACS analysis of the immune cell content in livers from naïve and B16LS9 tumor-bearing mice after entolimod treatment. This showed that entolimod treatment increased the percentage of NK cells in the livers of both naïve and tumor-bearing mice. The NK cell response elicited by entolimod was not due to proliferation of NK cells in the liver, but rather blood-borne homing of new cells to the tissue. In addition to promoting recruitment of NK cells to the liver, entolimod stimulated maturation (CD49b marker) and differentiation (CD11b and CD43 or CD27 markers) of NK cells equipped with effector function in the liver [37, 38]. CD27 is a key marker of the NK cell lineage, bisecting the mature NK cell pool into two functionally distinct subsets: the NK cell subset with low CD27 has a higher threshold for stimulation and appears to be tightly regulated by the expression of NK cell inhibitory receptors, while the NK cell subset with high expression of CD27 displays a greater effector function, exhibits a distinct tissue distribution and responsiveness to chemokines, and interacts with dendritic cells [39]. Our results showed a significant increase in the more highly developed CD27^+^CD11b^+^ NK cell population in the mouse liver after entolimod treatment (compared to vehicle treatment) and a reduction in the less developed CD27^−^CD11b^−^ and CD27^+^CD11b^−^ subsets (Figure 5A). Since CD27^−^CD11b^−^ and CD27^+^CD11b^−^ subsets act as precursors capable of giving rise to effector cells when needed [37], we conclude that entolimod stimulated these two less developed NK cell populations to differentiate into CD11b^+^CD27^+^ NK cells with effector function [38, 40]. Interestingly, CD11b^+^CD27^−^ NK cells, which are highly developed but lack effector function, are not significantly affected by entolimod treatment.

The changes in NK cell phenotype observed following entolimod treatment are consistent with activity of this cell population playing a role in the drug's anti-metastatic effects. This was demonstrated to indeed be the case by comparing the effects of entolimod treatment on B16LS9 UM liver metastasis in NK cell depleted (anti-asialo GM1 antibody-treated) versus NK cell sufficient (isotype control antibody-treated) mice (Figure 6). This experiment clearly showed that the inhibitory effect of entolimod on growth of B16LS9 metastases in the liver requires NK cells while also revealing a basal level of entolimod-independent NK cell-mediated antitumor activity. The involvement of NK cells in the activity of entolimod in the UM model is in line with our earlier studies demonstrating that entolimod treatment suppressed liver metastasis of mouse 4T1 breast and CT26 colon tumors via an NK cell-dependent mechanism [20].

In contrast to what was observed for NK cells, entolimod treatment did not have any significant impact on the level of NKT (CD3^+^NK1.1^+^) cells in the mouse liver (Figure 5B). Although these cells share some properties with NK cells (e.g., cytolytic activity), NKT lymphocytes are considered part of the adaptive immune system since they express a T-cell receptor. Adaptive immune responses differ from innate immune responses in that they are not immediate, requiring 3–5 days for clonal expansion and differentiation of antigen-specific effector lymphocytes [41], and in that they display “memory” resulting in more rapid and robust responses upon subsequent encounters with a given antigen. NKT cells, in contrast to NK cells, respond to antigen presented by the atypical MHC Class I molecule. Our finding that entolimod did not stimulate NKT cell fractions is consistent with the low MHC-I expression of B16LS9 tumor cells [34].

The *in vitro* study of CT activity of lymphocytes confirmed the critical role in entolimod mediated tumor cell elimination by demonstrating the inhibited cytotoxicity of hepatic lymphocytes isolated from mice after depletion of NK cells with anti-asialo GM1 antibody with and without entolimod treatment against B16LS9 tumor target cells. It is important to note that innate CT activity of both hepatic and splenic lymphocyte populations against B16LS9 tumor cells *in vitro* was lower in B16LS9 tumor-bearing mice than in naïve (non-tumor-bearing) mice suggesting the development of tumor-associated immunosuppression. Entolimod specifically restored and, in fact, increased the antitumor lymphocyte function in the liver, but not in the spleen. This finding provides further support for the key role of hepatocytes in entolimod-elicited antitumor immune responses.

In summary, this study demonstrates that the TLR5 agonist entolimod potently suppresses hepatic metastasis in the mouse B16LS9 ocular melanoma model when administered before or after tumor cell inoculation. Entolimod treatment stimulated mobilization of NK cells to the mouse liver and promoted their maturation and activation. Antibody mediated depletion of NK cells from mice completely abrogated the antimetastatic effect of entolimod *in vivo* and eliminated *in vitro* antitumor cytotoxic activity from hepatic lymphocyte populations. Together with other published studies demonstrating sensitivity of uveal melanoma to NK cell cytotoxicity in mouse and human xenograft models [34, 42], this work suggests that entolimod could be used clinically to effectively prevent and treat liver metastases arising from ocular melanoma due to its potent effects on NK cells in the liver.

## MATERIALS AND METHODS

### Mice

C57BL/6 mice were purchased from Jackson Laboratories (Bar Harbor, ME). C57BL/6-Tg(UBC-GFP) 30Scha/J mice (referred to as GFP mice) were bred and maintained at RPCI. The experiments with tumor-free mice were performed at RPCI and followed protocols approved by the RPCI Institutional Animal Care and Use Committee (IACUC). All experiments with the B16LS9 spontaneous metastatic model were conducted at EMORY according to the Guiding Principles in the Care and Use of Animals and conformed to the ARVO Statement for the Use of Animals in Ophthalmic and Vision Research.

### Tumor cells

The mouse B16LS9 melanoma cell line was kindly provided by Dario Rusciano, Friedrich Miescher Institute, Basel, Switzerland. These cells express high levels of c-Met and spontaneously metastasize to the liver [43]. B16LS9 cells were maintained in RPMI 1640 medium (MediaTech, Manassas, VA) supplemented with 10% fetal bovine serum (Hyclone, Logan, Utah), 1% L-glutamine (Hyclone), 2.5% HEPES (Lonza, Walkersville, MD), 1% sodium bicarbonate (Hyclone), 1% MEM essential vitamins (Hyclone), and 1% penicillin/streptomycin (Hyclone) in a 75-cm^2^ tissue culture flask (T-75; BD Biosciences, Franklin Lakes, NJ) in a CO_2_ incubator (Kendro, Asheville, NC) at 37°C with 5% CO_2_. For inoculation into mice, a suspension of B16LS9 cells was prepared in phosphate buffered saline (PBS) just prior to inoculation.

### Reagents

Entolimod was provided by Cleveland BioLabs, Inc. (Buffalo, NY). Entolimod stock solution (1 mg/ml in PBS) was stored at −80°C and diluted with sterile PBS containing 0.1% Tween 80 (PBS-T) to a final concentration 0.01 mg/ml for treatment of mice by s.c. injection. Entolimod was diluted in complete cell culture medium for *in vitro* use. Tumor necrosis factor-alpha (TNF) was ordered from PeproTech (Rocky Hill, NJ) and used as a positive control for detection of NF-κB activation using by the p65 nuclear translocation immunofluorescence assay.

### *In vivo* model of hepatic metastasis from intraocular B16LS9 tumors

B16LS9 cells were inoculated into the posterior compartment of the right eye using a transcorneal technique that allows the inoculated cells to remain in the eye [34, 44]. For each inoculation, 5 × 10^5^ cells were delivered in a volume of 2.5 μl. The mice were anesthetized with intraperitoneal injection of the ketamine/xylazine mixture and a tunnel was prepared from the limbus within the cornea, sclera, and ciliary body to the choroid with a 30-gauge needle under the guidance of a dissection microscope. The tip of a 10 μl glass syringe with a blunt metal needle (Hamilton, Reno, NV) was used to introduce 2.5 μl of cell suspension (5 × 10^5^ cells) into the posterior compartment through the needle track. The right eye was enucleated (removed) 7 days after tumor cell inoculation [45].

### Entolimod treatment

Each mouse received 1μg of entolimod in PBS containing 0.1% Tween 80 (PBS-T) in an injection volume of 100 ml or an equal volume of PBS-T (vehicle control) by subcutaneous injection, repeated every 3 days. Three groups of mice (*n* = 10 /group) were treated with entolimod with different times of treatment initiation relative to tumor cell inoculation: (i) 1 day before tumor cell inoculation (7 total injections), (ii) on the day of tumor cell inoculation (7 total injections), or (iii) on the 4th day after tumor cell inoculation (6 total injections). A 4th group of 10 mice was given vehicle (PBS-T) injections starting 1 day before tumor cell inoculation, as a control group. Mice were euthanized and livers were harvested 21 days after tumor cell inoculation.

For the experiment testing the effect of NK cell depletion on entolimod's efficacy against B16LS9 liver metastasis, mice were inoculated in the right eye with B16LS9 tumor cells followed by enucleation and entolimod/vehicle treatment starting on the day of tumor cell inoculation (7 total injections). In addition, however, the mice received intraperitoneal (i.p.) injections of anti-asialo GM1 antibody or rabbit IgG isotype control antibody (100 μg/200 μl per injection, Wako Laboratory Chemicals, Richmond, VA), as previously described [20]. The antibody were injected 24 hours before tumor cell inoculation, 30 minutes before the first entolimod/vehicle injection, and every 4 days thereafter (8 injections total).

For entolimod treatment of naïve (non-tumor-bearing) mice, a single s.c. injection of entolimod (1 μg in 100 ml volume) was administered to each mouse.

### Histological analysis of enucleated eyes and lungs and livers collected from B16LS9 tumor-bearing mice

Enucleated eyes removed from mice 7 days after B16LS9 tumor cell inoculation were processed for light microscopic examination. The eyes were fixed in formalin, embedded in paraffin blocks and sectioned. Serial 5-μm-thick sections were stained with hematoxylin-eosin (H&E) and evaluated for the presence and location of the melanoma. The section with the largest tumor area in each eye was photographed at x4, x10 and x40 magnification (Olympus BX41; Olympus, Tokyo, Japan). The tumor size was measured with ImageJ software (National Institutes of Health, Bethesda, MD).

After enucleation, the mice were treated with vehicle or entolimod as described and then euthanized 21 days after tumor cell inoculation. The livers and lungs were collected from each mouse. The livers and lungs were grossly examined, submerged in 4% neutral buffered formaldehyde, and processed for light microscopic examination. Three sections through the center of each liver and one section of each lung were microscopically evaluated (Olympus) for the presence of metastases, the average number of metastases per liver (average for three sections) and the number of lung metastases was determined, as previously described [46, 47].

### *In vitro* cytotoxicity assay

Liver and spleen lymphocytes were isolated using Lympholyte-M cell separation medium (Cedarlane, Hornby, Ontario, Canada) for use as effector cells in *in vitro* cytotoxicity (CT) assays. CT was assessed using the DELFIA^®^ EuTDA Cytotoxicity kit (PerkinElmer, Waltham, MA). Target cells (B16LS9 tumor cells) were loaded with a fluorescence-enhancing ligand (BATDA, bis (acetoxymethyl) 2,2′:6′,2′′-terpyridine-6,6′′ -dicarboxylate) for 30 minutes at 37°C, then washed and co-incubated with effector cells at 12.5:1, 25:1, 50:1 lymphocyte-to-target cell ratios in V-bottom 96-well-plates in a total volume of 200 μl of RPMI medium 1640 without phenol red. Control wells contained target cells with no effector cells (“spontaneous release”) or the lysed target cells by supplemented lysis buffer for maximum fluorescence release. CT was determined after 2 hours of incubation at 37°C by measuring the fluorescence released in 20 μl of supernatant harvested from each well using a fluorescence microplate reader. The percentage of specific lysis was calculated as follows:

% Specific release = [(Experimental release− Spontaneous release) / (Maximum release − Spontaneous release)] × 100.

### NK cell isolation and flow cytometry

For analysis of NK cells in naïve C57BL/6 mice, livers were collected at the indicated times post-treatment by *in vivo* perfusion with a solution of 0.5 μM EGTA (Sigma-Aldrich) containing 0.2 mg/mL collagenase type IV (Worthington Biochemical, Lakewood, NJ) followed by mechanical disruption and *in vitro* digestion for 30 minutes at 37°C. Cells were then passed through a 70 μM cell strainer. Single cell suspensions were stained for 20 minutes at 4°C with the following cocktails of monoclonal antibodies (mAbs): (1) Pacific Blue CD45 (clone 30–F11), Brilliant Violet (BV) 711 CD3ε (clone 145–2C11), PE-Cy5 NK1.1 (clone 29A1.4); (2) Pacific Blue CD45 (clone 30–F11), BV711 CD3ε (clone 145–2C11), PE-Cy5 NK1.1 (clone 29A1.4), PE-Cy7 CD49b (clone DX5), Ax700 CD11b (clone M1/70), PE CD43 (clone 1B11); (3) Pacific Blue CD45 (clone 30–F11), BV711 CD3ε (clone 145–2C11), PE-Cy5 NK1.1 (clone 29A1.4), PE-Cy7 CD49b (clone DX5), PE FasL (clone MFL3), BV605 CD69 (clone H1.2F3). For the experiment involving NK cell proliferation by BrdU incorporation, 1 mg BrdU was administered i.p. 2 h prior to collection and processing of livers for FACS. Cells were stained with antibodies in pane 1 in addition to an anti-BrdU antibody according to manufacturer's instructions (BD Biosciences).

For NK cell analysis in UM tumor-bearing mice, livers and spleens were excised from entolimod/PBS-T treated mice (started on the day of tumor cell inoculation, as described above) 21 day after tumor cell inoculation and homogenized with glass slides. Cells were gently forced through a sterile 70-μm nylon cell strainer (BD Biosciences, Bedford, MA) with a sterile syringe. Viable lymphocytes were isolated from the cell suspension by density separation using Lympholyte-M cell separation medium. After centrifugation at 1500 × g for 20 minutes at room temperature, the second layer of cells (containing lymphocytes) was carefully removed, diluted with fresh medium and centrifuged at 800 x g for 10 minutes, washed three times in the medium, counted, and used for staining with cell type-specific markers. CD3^−^ NK cells were identified by positive staining with FITC anti-mouse NK1.1 (aka NKR-P1C, LY-55; PK136) (BD Pharmingen, San Diego, CA), PE anti-mouse pan-NK cells (BD Pharmingen, San Diego, CA) and anti-mouse CD3 (17A2) (eBioscience, San Diego, CA). The activation status and maturation stage of NK cells were determined by staining the cells with mAbs against CD11b (M1/70.15) (Invitrogen, Camarillo, CA) and CD27 (LG.7F9) (eBioscience, San Diego, CA). Data acquisition and analysis were performed using a FACSAria instrument (BD Biosciences, San Jose, CA) and FlowJo software (Tree Star, Ashland, OR), respectively.

### NK cell homing

Naïve splenocytes were collected from GFP mice by mechanical disruption of spleens through a 40 μM cell strainer followed by adoptive transfer via i.v. injection into naïve C57BL/6 mice immediately prior to entolimod or PBS treatment. Livers were harvested 5, 24, and 120 h after treatment by *in vivo* perfusion followed by *in vitro* digestion to assess presence of adoptively transferred NK cells (CD45^+^ GFP^+^ CD3ε^−^ NK1.1^+^) similarly as above by FACS analysis.

### *In vitro* p65 translocation assay

B16LS9 cells were cultured under standard conditions and treated with 100 ng/ml entolimod, 10 ng/mL TNF (positive control), or PBS (negative control) for 30 minutes at 37°C. For immunofluorescence staining, cells were fixed with 10% paraformaldehyde for 5 min at room temperature, then washed 3 times with PBS and incubated with auto-fluorescence blocking solution containing 2% glycine, 0.2% Tween 20, 0.2% Triton X-100 in PBS (1 h, RT). The cells were then stained by the indirect immunofluorescence method with 1:200 rabbit anti-p65 (Cell Signaling, Danvers, MA), followed by 1:500 donkey anti-rabbit Alexa Fluor^®^ 488 (Jackson Immuno Research Laboratory, West Grove, PA) and 1:100 anti-phalloidin Alexa Fluor^®^ 647 (Invitrogen, Camarillo, CA). All antibodies were diluted in blocking solution containing 5% normal donkey serum, 0.2% Triton X-100, and 0.2% Tween 20 in PBS. After staining, the cells were mounted with ProLong Gold antifade reagent with DAPI (Invitrogen, Camarillo, CA) and viewed under a confocal microscope. The DAPI counterstain permits visualization of DNA in the blue channel and was used to verify nuclear localization of p65.

### Statistical analysis

The two-tailed unpaired Student's *t*-test was applied to determine whether the number and size of metastatic melanoma nodules, amount of immune cells and lymphocyte cytotoxic activity differed significantly between vehicle-treated and entolimod-treated groups (GraphPad Prism software). *P* values ≤ 0.05 were considered statistically significant.

## SUPPLEMENTARY MATERIAL FIGURES


