# Supplementary material for: The Toll-like receptor 5 agonist entolimod suppresses hepatic metastases in a murine model of ocular melanoma via an NK cell-dependent mechanism
Source: Oncotarget. 2015 Dec 8;7(3):2936–50. doi: 10.18632/oncotarget.6500 (PMC4823082; doi:10.18632/oncotarget.6500)
Supplement: Supplementary file 1 [file oncotarget-07-2936-s001.pdf]

The Toll-like receptor 5 agonist entolimod suppresses hepatic metastases in a murine model of ocular melanoma via an NK cell-dependent mechanism

Supplementary Materials

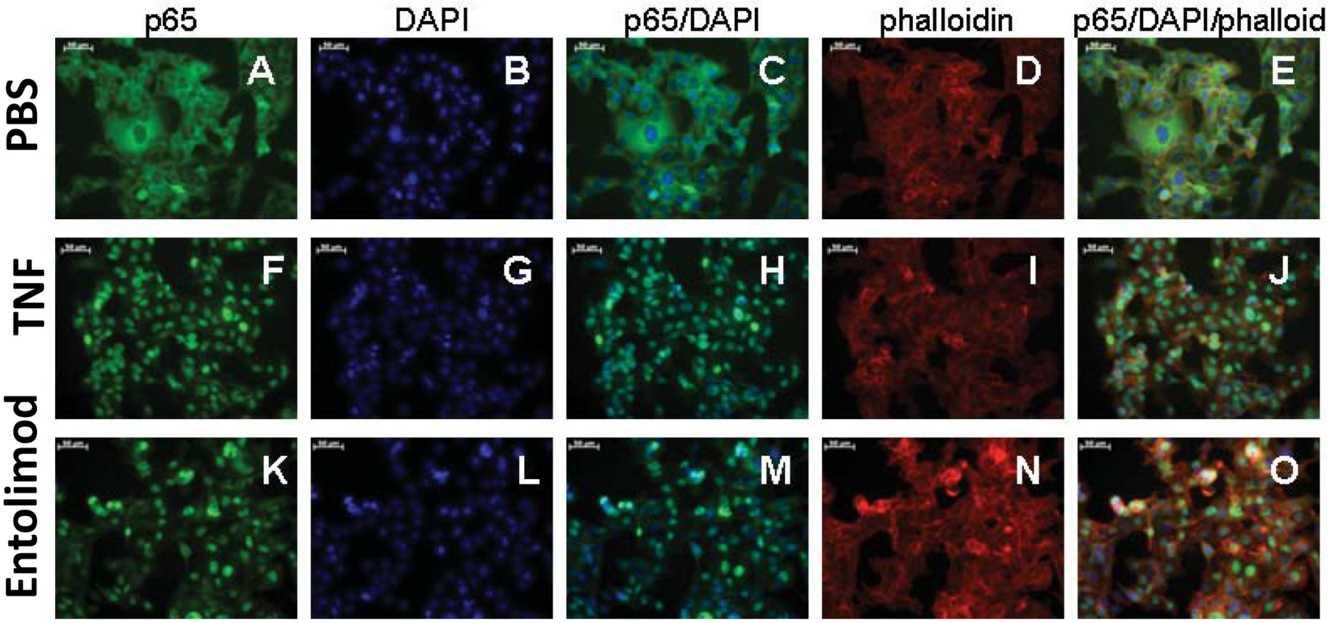

Supplementary Figure S1: Immunohistochemical detection of nuclear translocation of the p65 subunit of NF-kB following *in vitro* treatment of B16LS9 cells with entolimod. Representative fluorescent microscopic images of B16LS9 cells treated with PBS (**A**–**E**), TNF (10 ng/ml) (**F**–**J**), or entolimod (100 ng/ml) (**K**–**O**) for 30 minutes and then stained with monoclonal rabbit anti-p65 antibody followed by Alexa Fluor 488-labeled anti-rabbit IgG antibody (green), Alexa Fluor 594-labeled phalloidin (actin stain, red), and DAPI (DNA stain, blue).

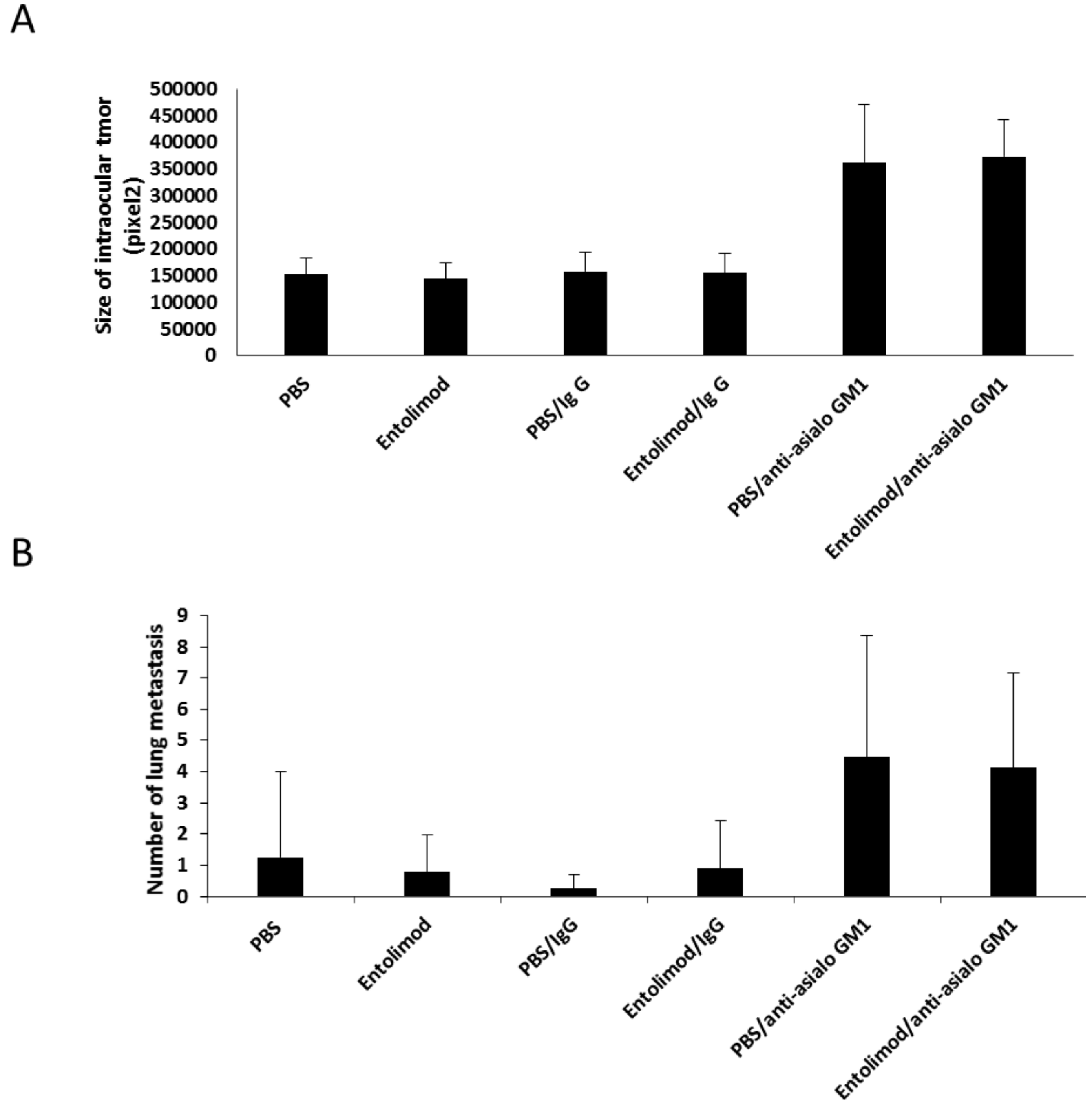

Supplementary Figure S2: Effect of entolimod treatment with or without NK cell depletion on growth of B16LS9 tumors in the eye and metastasis to the lung. C57BL/6 mice were inoculated with B16LS9 cells in the choroid of the right eye and treated with vehicle (PBS-T), entolimod, PBS-T with control IgG antibody, entolimod with control IgG antibody, PBS-T with anti-asialo GM1 antibody or entolimod with anti-asialo GM1 antibody (see text for details on treatments). (**A**) The mean size (area in $pixel^2$) of intraocular tumors was determined by microscopic evaluation of serial H & E-stained sections of tumor-bearing eyes enucleated 7 days after tumor cell inoculation ($n = 6$ mice/group). Error bars indicate SEM. (**B**) The mean number of lung metastases per lung was determined on Day 21 after tumor cell inoculation ($n = 6$ mice/group). Metastatic foci were counted under a microscope in one H & E-stained section from each lung (two per mouse). Differences in the area of intraocular tumor ($pixel^2$) and number of lung metastases between entolimod-treated and corresponding control groups were not significant ($p > 0.05$); however, both intraocular tumor growth and lung metastasis was significantly higher in groups treated with anti-asialo GM1 antibody than in those treated with control antibodies (with or without entolimod) or those treated with PBS-T or entolimod alone ($p < 0.02$ for all pair-wise comparisons).

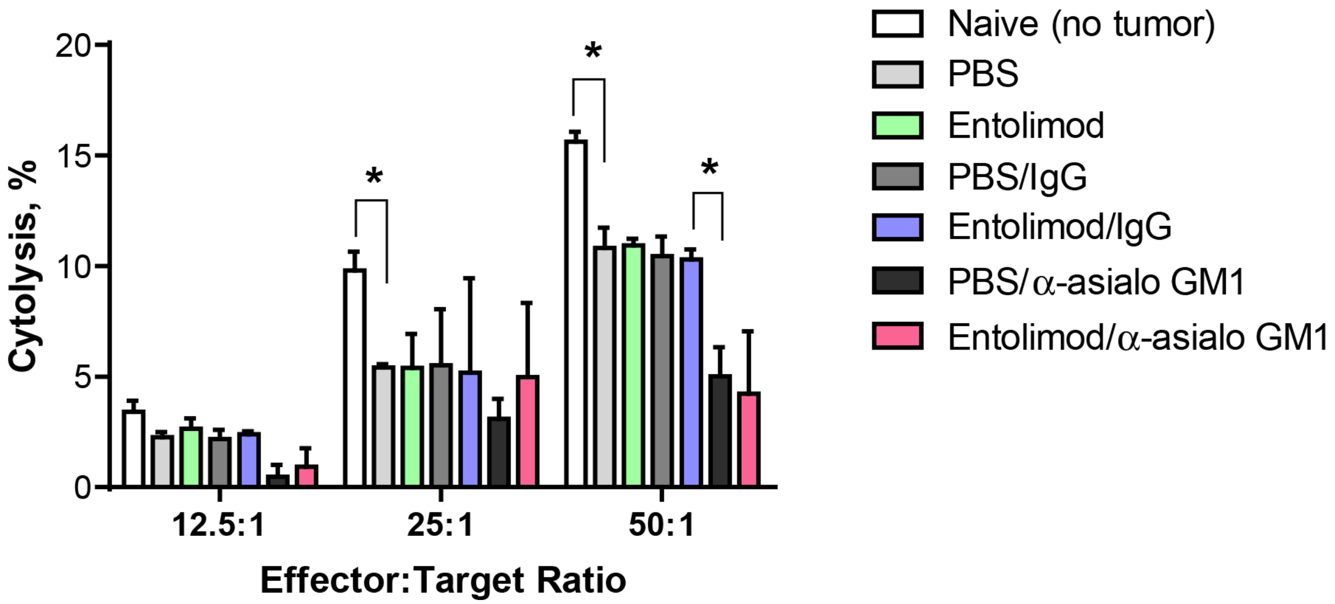

Supplementary Figure S3: Effect of entolimod treatment with or without NK cell depletion on *in vitro* cytotoxicity (CT) of splenic lymphocytes towards B16LS9 tumor cells. C57BL/6 mice were inoculated with B16LS9 melanoma cells in the choroid of the right eye and treated with vehicle (PBS-T), entolimod, PBS-T with control IgG antibody, entolimod with control IgG antibody, PBS-T with anti-asialo GM1 antibody or entolimod with anti-asialo GM1 antibody (see text for details on treatments). Naïve (non-tumor-bearing) mice were used as an additional control. Lymphocytes were purified from spleens collected from the mice 21 days after tumor cell inoculation and incubated with B16LS9 target cells at effector-to-target cell ratios of 12.5:1, 25:1 and 50:1. The mean % cytolysis ± SD is presented, 6 mice/group. Asterisks indicate significant differences ($p \leq 0.05$ by Student's *t*-test) between the indicated groups.
